# Development and Validation of a Family Caregiver Constraint Index

**DOI:** 10.1001/jamanetworkopen.2026.15350

**Published:** 2026-05-28

**Authors:** Jennifer Tjia, Emmanuella Asiedu, Jonggyu Baek, Kate Lapane, Arlene Ash, Julie Flahive, Susan DeSanto-Madeya, Chengwu Yang

**Affiliations:** 1 Department of Population and Quantitative Health Sciences, UMass Chan Medical School, WorcesterMassachusetts; 2 Department of Population and Quantitative Health Sciences, & Psychometrician of the Measurement, Outcome, and Design Section, UMass Chan Medical School, Worcester, Massachusetts; 3 Elisabeth DeLuca School of Nursing, University of Connecticut, Storrs; 4 Department of Population Health, Schmidt College of Medicine, Florida Atlantic University, Boca Raton

## Abstract

**Importance:**

Individuals with acute, chronic, and life-limiting illness often rely on family caregivers to navigate health care, but caregivers’ ability to spend time providing support may be limited by socioeconomic constraints, such as inflexible work hours.

**Objective:**

To develop and validate the Family Caregiver Constraint Index (FCCI), a summary measure of area-based factors that can affect synchronous caregiver engagement with health care systems.

**Design, Setting, and Participants:**

This cross-sectional study used 2017 to 2021 American Community Survey 5-year estimate data for 32 789 populated US 5-digit zip code tabulation areas (neighborhoods) and selected candidate indicators relevant to caregiving-related domains. Initial variable selection was guided by engagement with community members with chronic disease and/or family caregiver experience. Analyses were conducted between June 2023 and July 2025.

**Main Outcomes and Measures:**

Principal components analysis identified and quantified distinct subindices and correlations among indicators, which were used to construct scores for subindices and create a standardized summary FCCI score. The association between the FCCI and hospital-based health care outcomes from the Dartmouth Atlas of Health Care was examined using crude and adjusted linear regression models that accounted for year and state in which the HSA is located.

**Results:**

The American Community Survey sample from which the FCCI is developed draws from the US population of 329 725 481 persons, of whom 166 518 866 (50.5%) are female and 52 888 621 (16.0%) are aged 65 years or older. The 13-item FCCI mapped to 4 subindices: competing demands (work and childcare responsibilities, 2 items), long-distance work commute (2 items), income constraint (5 items), and recent immigration (4 items). Higher FCCI scores were associated with greater inpatient days (increase of 0.80 day; 95% CI, 0.71-0.89 day; *P* < .001) and intensive care unit and/or coronary care unit days (increase of 0.48 day; 95% CI, 0.40-0.56 day; *P* < .001) during the last 6 months of life, higher percentage of in-hospital deaths (increase of 1.06%; 95% CI, 0.79%-1.34%; *P* < .001) and percentage of deaths that included an intensive care unit and/or coronary care unit admission (increase of 1.27%; 95% CI, 0.99%-1.54%; *P* < .001).

**Conclusions and Relevance:**

In this cross-sectional study of barriers to family caregiver engagement, the FCCI ranked neighborhoods based on area-level factors that adversely impact the time available for family caregivers to be synchronously present for their care recipient’s health care visits. Future research should examine the relationships among FCCI scores, caregiver engagement, hospital care, caregiver well-being, and patient health outcomes.

## Key Points

**Question:**

Is an area-based index of barriers to family caregiving associated with hospital-based health outcomes?

**Findings:**

This cross-sectional study of an index of barriers to family caregiving used data for 329 725 481 persons from the 2017 to 2021 American Community Survey 5-year estimates. The 13-item Family Caregiver Constraint Index mapped to 4 subindices: competing demands (work and childcare responsibilities), long-distance work commute, income constraint, and recent immigration, with higher scores indicating greater barriers to synchronous family caregiving.

**Meaning:**

Preliminary testing of the Family Caregiver Constraint Index suggests the validity of this index to measure caregiving-related domains that can be barriers to family caregiver engagement and their association with care recipient health outcomes.

## Teaser

This cross-sectional study reports the development and validation of the Family Caregiver Constraint Index, a neighborhood-based index measuring factors potentially affecting caregiver engagement with health care systems.

## Introduction

An estimated 50 to 60 million family and friends are caregivers to loved ones in the US.^1,2^ They are frequently unpaid and can be any relative, partner, or friend who has a personal relationship with, and significantly assists, a person with a disabling condition in everyday activities.^2,3^ These individuals, hereafter *family caregivers* or *caregivers*, are a crucial element of the health care system and engage with clinicians in ways that affect patient care. Caregivers make appointments (67%), speak to clinicians (60%),^4^ and engage health care professionals in conversations that clarify patient values.^5^ They also navigate and coordinate care across multiple clinicians and reduce care fragmentation.^5,6^ Caregivers can be advocates who monitor symptoms, provide emotional and spiritual support, and help with transportation.^4^ Yet a caregiver’s ability to engage with the health care system is limited by their personal circumstances and available resources.^7^

Uncompensated caregiving is not free. On average, caregivers provide 20 hours of service a week, which can crowd out paid employment.^2,8^ Up to 20% retire early or quit their job,^9^ 22% exhaust short-term savings, 20% have late or unpaid bills, and 11% are unable to afford basic expenses like food.^2^ These stresses can affect a caregiver’s ability to provide care, take care recipients to medical appointments, and engage with clinicians during acute hospitalizations. For example, lack of access to affordable and reliable transportation makes going to medical appointments difficult and time consuming.^10^ On the other hand, caregivers with greater personal resources and disposable time are likely to be more available for synchronous communication with clinicians, possibly contributing to better health outcomes for care recipients. Thus, it is important to measure the impact of barriers to family caregivers on patient health outcomes,^3^ as well as to quantify the positive impacts of caregiving on health outcomes.

There are several measures of neighborhood-based resource availability, including the Area Deprivation Index,^11^ the Childhood Opportunity Index,^12^ the Neighborhood Stress Score,^13^ the Social Deprivation Index,^14^ and the new Structural Racism Effect Index.^15^ These population-specific indices of geographic opportunity^16,17^ demonstrate that associations between neighborhood context and health outcomes matter tremendously. There are also individual-level, self-administered, measures of caregiver burden and engagement including the Zarit Caregiver Burden Interview^18^ and the Caregiving Health Engagement Scale.^19^ However, these are not designed for neighborhood-level measurement of structural barriers to caregiving.

To address this gap, we developed a Family Caregiver Constraint Index (FCCI) that aims to facilitate equity analyses of health outcomes for the care recipient by area-based constraints on caregivers’ ability to play a supporting role in health care. The index captures institutional, neighborhood, and cultural factors that can affect real-time information sharing and decision-making for at-risk patients. The FCCI is designed to enable a research focus on the impact of neighborhood constraints on family caregiver engagement and, ultimately, patient outcomes.^20,21^

## Methods

This cross-sectional study of secondary data was reviewed and approved by the UMass Chan Medical School institutional review board. Written informed consent was not required for this study using deidentified data. The study followed the Strengthening the Reporting of Observational Studies in Epidemiology (STROBE) reporting guideline. Analyses were conducted between June 2023 and July 2025.

### Data Sources

We constructed the FCCI using data from the 2017 to 2021 American Community Survey (ACS). The ACS is an ongoing national annual survey from the Census Bureau^22,23^ that collects information relevant to health research such as self-reported age, race and ethnicity, marital status, educational attainment, health insurance, employment status and location, commute to work, vehicles available, household income, food assistance benefit, ownership and/or home value and/or rent, owner housing costs, computer and internet use, ancestry, place of birth, citizenship, and language spoken at home. Self-reported race and ethnicity in the ACS is reported as American Indian or Alaskan Native, Asian, Black or African American, Hispanic or Latino, Native Hawaiian and Pacific Islanders, some other race alone, or 2 or more races. Data on race and ethnicity are included in this study because caregiving affects individuals differentially by racial and ethnic background. The 2017 to 2021 ACS 5-year estimates are based on a total sample size of approximately 17.5 million addresses collected over 60 months.

### Variable Collection and Selection

On the basis of a literature review, clinical expertise, and the lived experience of persons with chronic disease and family caregiving, we conceptualized factors that support a family caregiver’s being able to assist a care recipient. We sought to identify neighborhood-level caregiver resources that affect the ability to go to the hospital during the work week to communicate with clinicians. Socioeconomic variables measured at the neighborhood level capture aspects of community stratification, opportunity structures, and social conditions.^20,21,24,25,26^ Investigators met with community partners and caregivers with lived experience of caregiving during 2, 90-minute, hybrid (in-person and video-conferenced) meetings and reviewed 98 candidate variables that mapped to domains reflecting barriers to caregivers’ ability to synchronously engage with clinicians during an acute hospitalization. We collaboratively selected 29 candidate variables for inclusion (eTable 1 in Supplement 1). We then collapsed closely related candidate variables (eg, has computer with dial-up internet alone and has a computer with a broadband internet), resulting in 13 composite variables: 2 for transportation (no car and public transport); 3 for work commute (work outside of home, employment outside of county, and long travel time to work); 2 for communication and acculturation (language other than English at home and noncitizen); 2 for financial constraints (income constrained and burden of monthly housing cost); and 1 each for internet connectivity (inability to teleconference), employment (2 or more working persons in a household), food stability (food assistance use), and childcare demand (household with children). We characterized each composite variable by the percentage of residents reporting its presence at the Zip Code Tabulation Area (ZCTA) level.^22^

Since time availability is a privilege,^27^ we oriented all variables in the same direction to make higher values indicate a time disadvantage due to greater competing demands or reliance on public transportation. Recognizing that some variables affected both material resources (eg, finances) and personal stress (eg, time availability)—and sometimes in opposite directions—we used the lens of time availability to operationalize variable’s direction. For example, we coded “employment status—2 or more working people in the household” to reflect the demand on time as a disadvantage, even though this could have been coded to reflect material advantage through higher earnings.

### Internal Structure, Domain Identification, and Index Construction

Principal components analysis (PCA) was used to examine the relationships and internal structure of the proposed index variables. We included data from 32 789 of the 33 774 US ZCTAs, excluding the 985 ZCTAs with either 0 population or missing values on key candidate variables. Kaiser-Meyer-Olkin and Bartlett test examined the appropriateness of these data for PCA, with a Kaiser-Meyer-Olkin value greater than 0.5 indicating adequate sampling and a Bartlett test with *P* < .05 confirming that variables were adequately correlated for PCA. We interpreted and labeled each of the components based on variable loadings greater than 0.4. The FCCI was calculated by first summing the scores from each of the PCA-identified subindices and then standardizing that sum to create a population-weighted standardized ZCTA FCCI score (eAppendix in Supplement 1).

### Index Validation

We examined face validity by categorizing ZCTAs into deciles of FCCI scores for the outcome ZTCA’s proportion (percentage) of population self-reporting as American Indian or Alaskan Native, Asian, Black or African American, Hispanic or Latino, Native Hawaiian and Pacific Islander, some other race alone, or 2 or more races in the ACS. We then explored the association between FCCI and aggregated health care outcomes from the 2017 to 2018 Dartmouth Atlas Project aggregated at the level of health service areas (HSA).^28^ HSA-level data are derived from health care utilization trends, allowing for the analysis of empirical health care–seeking patterns. HSAs are a collection of zip codes whose residents receive most of their hospitalizations from hospitals in that area. Most HSAs contain only 1 hospital. We derived HSA-level FCCI by calculating the average ZCTA-level FCCIs for a given HSA, using the population-weighted standardized ZCTA FCCI. We chose health care service outcomes plausibly affected by caregiver engagement, including (1) inpatient days per decedent during last 6 months of life, (2) intensive care unit (ICU) or coronary care unit (CCU) days per decedent during last 6 months of life, (3) percentage of in-hospital Medicare deaths, (4) percentage of decedents admitted to ICU and/or CCU during the hospitalization in which death occurred, and (5) hospital and skilled nursing facility reimbursement per enrollee. We expected that higher FCCI scores would be associated with poorer outcomes (eg, higher percentage of in-hospital deaths).

### Statistical Analysis

We examined associations between the FCCI and aggregated HSA-level health care outcomes in crude and adjusted linear regression models that accounted for year and state in which the HSA is located. Analyses included data from at least 1 of 3436 HSAs in the US. We excluded HSAs that were too small for statistical precision or suppressed for privacy reasons. Sensitivity analysis further explored the relationship of FCCI with the Structural Racism Effect Index, another neighborhood-level index that quantifies of the impact of structural racism,^15^ using crude and adjusted linear regression models that accounted for proportion ZCTA population male, age older than 65 years, and Asian, Black and African American, Hispanic and Latino, and other race and ethnicity. *P* values were based on 2-tailed tests of statistical significance. PCA and linear regressions were performed using SAS version 9.4 (SAS Institute); all other statistical analyses were performed in Stata version 13 (StataCorp).

## Results

### Descriptive Statistics

The total weighted population for this study included 329 725 481 individuals, of whom 166 518 866 (50.5%) were female, 52 888 621 (16.0%) were aged 65 years or older, and 196 010 370 (59.4%) self-identified as White. The demographic characteristics of the 2017 to 2021 ACS 5-year population estimates are in eTable 2 in Supplement 1.

### Principal Components Analysis

Preliminary data examination showed the Kaiser-Meyer-Olkin = 0.62 and Bartlett test *P* < .001, indicating appropriateness for PCA (eTable 3 in Supplement 1). Five components had an eigenvalue greater than 1 and cumulatively explained 64% of variation in the data (eTable 4 in Supplement 1). The scree plot shows eigenvalues leveling off at the third component (eFigure 1 in Supplement 1). Both the 5- and 4-component structure fit the data adequately (eTable 5 in Supplement 1). However, the 5-factor structure had a single-item domain (Childcare responsibilities) which is undesirable in index development (eTable 5 in Supplement 1). Since it is more desirable to have fewer components in applied research, we decided upon the 4-component structure. Table 1 shows the 4-component solution. Component 1 included 5 composite variables (teleconference inability; 2 or more working persons in the household; food assistance use; low income; and burden of monthly housing cost), each with a loading greater than 0.50. We label this component as *income constraint*. Component 2 included 4 composite variables (language other than English at home, noncitizen, no car, and public transport use), each with a loading of greater than 0.60. We label this component as *recent immigration*. Component 3 included 2 indicators (work outside the home and household with children), each with a loading of greater than 0.60. We label this component as *work and childcare responsibilities*. The final component included 2 indicators (employment out of county and long travel time to work), each with loadings greater than 0.70. We label this component as *long commute to work*.

**Table 1.** Loadings for Indicators Comprising the Family Caregiver Constraint Index: American Community Survey for US 2017 to 2021^a^

Indicators	Loading (interpretation)			
	Component 1 (income constraint)	Component 2 (recent immigration)	Component 3 (work and childcare responsibilities)	Component 4 (long commute to work)
Inability to teleconference	0.70^b^	−0.14	−0.18	0.08
≥2 Working persons in household	−0.58^b^	−0.09	0.05	0.06
Food assistance use	0.70^b^	0.06	0.28	0.13
Low income	0.88^b^	−0.11	−0.06	−0.01
Burden of monthly housing cost	0.52^b^	0.31	0.08	−0.15
Public transport use	0.02	0.76^b^	−0.16	0.19
No car	0.12	0.64^b^	−0.26	0.07
Language other than English at home	0	0.69^b^	0.41	−0.16
Noncitizen	−0.07	0.72^b^	0.37	−0.22
Work outside the home	−0.02	0.03	0.62^b^	−0.10
Household with children	0.01	−0.05	0.68^b^	0.18
Work out of county	−0.04	−0.18	0.08	0.76^b^
Long travel time to work	0.02	0.19	−0.04	0.73^b^

^a^ Observations include 32 789 zip code tabulation areas in the US for variables related to ability of caregivers to have time and resources to engage with sick family members.

^b^ Denotes variables with loading values greater than 0.40.

Table 2 shows the detailed distribution of FCCI scores based on a summary of components from the PCA. For each of the 4 components, we derived scores at the ZCTA level that represent the population prevalence of the composite variables for that geographic area. For example, within the component recent immigration, the composite variables represent the combined percentage of the population in the ZCTA who said yes to 4 composite variables (ie, language other than English, noncitizen, no car, and public transport use); the probability of each composite variable ranges from 0 to 1. Table 2 shows mean (SD) scores for each of the 4 components. The standardized FCCI scores ranged from −3.44 to 8.98; higher scores indicate greater caregiver constraint. Overall, 92% (n = 30 166) of the FCCI scores at the ZCTA-level fell between −2.0 and 2.0 (eFigure 2 in Supplement 1). The Figure shows the geographic variation of FCCI across the US.

**Table 2.** Distribution of Family Caregiver Constraint Index Scores Based on a Summary of Subindices From Principal Component Analysis

Subindices	Mean (SD)^a^	Median (IQR)^a^	Minimum	Maximum
Income constraint	0.48 (0.21)	0.48 (0.40 to 0.56)	0	4
Recent immigration	0.17 (0.24)	0.07 (0.03 to 0.21)	0	3.63
Work and childcare responsibilities	0.50 (0.29)	0.46 (0.27 to 0.70)	0	2.33
Long commute to work	0.36 (0.25)	0.33 (0.17 to 0.52)	0	2
Family Caregiver Constraint Index Standardized^b^	0 (1)	−0.45 (−1.00 to 0.19)	−3.44	8.98

^a^ The median and mean are different because the data are not normally distributed.

^b^ Family Caregiver Constraint Index higher scores indicate greater caregiver constraint.

**Figure.**
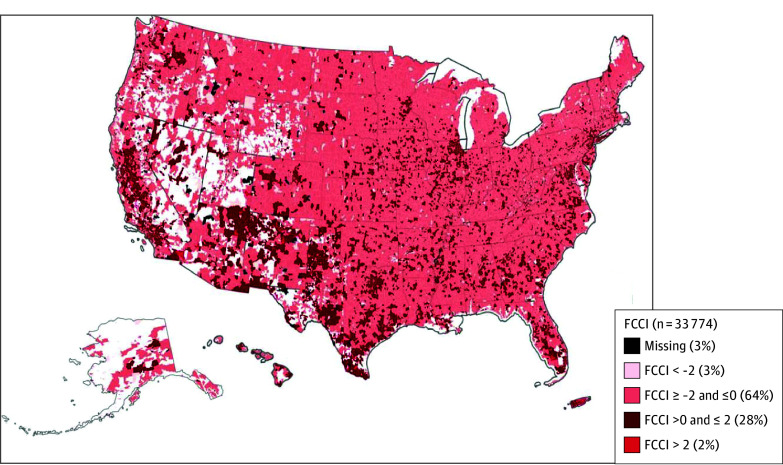
Map Depicting Geographic Distribution of Family Caregiver Constraint Index Across the US Map of the US shows the geographic distribution of Family Caregiver Constraint Index (FCCI). Higher scores indicate greater caregiver constraint. White areas are unpopulated.

Results from validity analysis show that deciles of FCCI were monotonically related to percentage of persons self-reporting as American Indian or Alaskan Native, Asian, Black or African American, Hispanic or Latino, Native Hawaiian and Pacific Islander, some other race alone, or 2 or more races in ZTCAs. FCCI decile 1 was associated with the mean (SD) lowest percentage of persons in this category 13.3% (17.4%) while FCCI decile 10 was associated with the highest percentage of persons in this category (mean [SD], 40.0% [28.0%] (Table 3). Additionally, in both unadjusted and adjusted regression models, the FCCI was significantly associated with HSA-level health care outcomes. After adjusting for year data was collected and state in which HSA is located, for every 1 SD increase in FCCI, we observed that (1) inpatient days per decedent during last 6 months of life increased by 0.80 day (95% CI, 0.71-0.89 day; *P* < .001), (2) ICU and/or CCU days per decedent during last 6 months of life increased by 0.48 day (95% CI, 0.40-0.56 day; *P* < .001), (3) Medicare deaths occurring in hospital increased by 1.06% (95% CI, 0.79%-1.34%; *P* < .001), (4) deaths that included ICU and/or CCU admission increased by 1.27% (95% CI, 0.99%-1.54%; *P* < .001), and (5) hospital and skilled nursing facility reimbursement per enrollee increased by $382.73 (95% CI, $321.19-$444.25; *P* < .001). All associations were statistically significant (Table 4). Sensitivity analysis shows a statistically significant association between Structural Racism Effect Index scores and FCCI scores (adjusted β = 0.10; 95% CI, 0.08-0.11; *P* < .001) (eTable 6 in Supplement 1).

**Table 3.** Racial and Ethnic Makeup Across the US by Decile of Family Caregiver Constraint Index at the ZCTA Level^a^

**Family Caregiver Constraint Index (deciles)**	Persons who self-reported being from a minoritized racial or ethnic group, %^b^	
	Mean (SD)	Median (IQR)
Decile 1	13.3 (17.3)	7.6 (1.7-17.6)
Decile 2	15.2 (16.5)	9.8 (4.3-20.8)
Decile 3	16.7 (17.3)	11.0 (4.8-23.1)
Decile 4	17.4 (18.2)	11.2 (5.1-23.6)
Decile 5	19.2 (19.4)	12.1 (5.6-27.4)
Decile 6	22.0 (21.4)	14.7 (6.0-32.0)
Decile 7	25.0 (23.7)	17.2 (6.4-37.5)
Decile 8	28.8 (26.2)	21.0 (7.8-43.6)
Decile 9	37.4 (31.0)	32.0 (9.6-56.0)
Decile 10	40.0 (28.0)	40.9 (14.6-61.7)
Increase in percentage of persons who self-reported being from a minoritized racial or ethnic group per decile	3.0 (2.3)	Not applicable

Abbreviation: ZCTA, Zip Code Tabulation Area.

^a^ The national total number of ZCTAs is 32 608. There are 3206 or 3207 ZCTAs in each decile.

^b^ Minoritized racial and ethnic groups include American Indian or Alaskan Native, Asian, Black or African American, Hispanic or Latino, Native Hawaiian and Pacific Islander, some other race alone, or 2 or more races.

**Table 4.** Unadjusted and Adjusted Associations Between Health Service Area Family Caregiver Constraint Index and Health Care Outcomes by Health Service Area

Outcomes	Estimate (95% CI)	*P* value
Inpatient days per decedent during last 6 mo of life, d (n = 3026 unique clusters)^a^^,^^b^		
Unadjusted	1.14 (1.03-1.24)	<.001
Adjusted^c^	0.80 (0.71-0.89)	<.001
ICU and/or CCU days per decedent during last 6 mo of life, d (n = 2719 unique clusters)^a^^,^^b^		
Unadjusted	0.79 (0.70-0.89)	<.001
Adjusted^c^	0.48 (0.40-0.56)	<.001
Medicare deaths occurring in hospital, % (n = 2010 unique clusters)^a^^,^^b^		
Unadjusted	1.76 (1.45-2.07)	<.001
Adjusted^c^	1.06 (0.79-1.34)	<.001
Deaths that included ICU and/or CCU admission, % (n = 1668 unique clusters)^a^^,^^b^		
Unadjusted	2.02 (1.75-2.29)	<.001
Adjusted^c^	1.27 (0.99-1.54)	<.001
Hospital and skilled nursing facility reimbursement per enrollee, $ (n = 3436 unique clusters)^a^^,^^b^		
Unadjusted	517.63 (440.53-594.72)	<.001
Adjusted^c^	382.73 (321.19-444.26)	<.001

Abbreviations: CCU, cardiac intensive care unit; ICU, intensive care unit.

^a^ The Dartmouth Atlas data (outcome variables) is based on Medicare claims data and enrollee counts (denominator data).

^b^ Analysis is based on 2017 and 2018 data.

^c^ All analyses for all outcomes used 2017 to 2018 data and are adjusted for year and state of the Health Service Area.

## Discussion

In this cross-sectional study developing and validating the FCCI, we show that this index captures the variation of resource limitations that impact caregivers’ time and ability to attend to family members with health care needs. The FCCI ranks neighborhoods according to area-level factors that adversely impact the time available for family caregivers to be synchronously present for care recipients’ health care visits. While the FCCI incorporates some standard measures of economic hardship such as income limitation, we meld these with domains impacting time available to caregivers such as competing demands for work. This is important because being able to consider caregiver engagement in terms of time as a resource is a step toward acknowledging that unpaid family caregiving is not free.

The FCCI captures key barriers to integrating family caregivers as meaningful partners in the health care team^3^ that were identified by RAND, including barriers to communication and information sharing, time limitations and competing demands, and cultural barrriers.^3^ We know of no other area-level family caregiver resource constraint measure applicable to population-level outcomes research.^15^ Other measures, such as the caregiver strain index,^29^ screen for factors contributing to caregiver strain after hospital visits. The Caregiving Health Engagement Scale measures caregivers’ psychological attitude to be an active, skilled and motivated player in the care process of loved ones,^19^ and the Zarit Burden Interview^18^ evaluates the personal stress, strain, and emotional impact of caregiving. In contrast, the FCCI quantifies structural barriers at the neighborhood level in a way that quantifies of the effects of structural barriers to caregiver engagement at the population level. Prior measures do not get at area-level barriers to a caregivers’ ability for synchronous communication with clinicians as a critical aspect of high-quality health care delivery.^30^

The adage that time is money is well known. Gee et al^27^ recently summarized evidence about time availability as a hidden determinant of health inequities. Time is a major social resource and is essential to health and human agency. Time allows for important activities such as exercise and social engagement. However, lack of time is associated with stress and illness.^31,32^ The literature about how time is a measurable social determinant of health that is shaped by racism across the life course is in its infancy, but the creation of the FCCI is a step forward. The FCCI operationalizes time as a resource and quantitatively measures time-dependent elements of caregiving that may affect health care delivery.

Most of the literature about the benefit for caregivers at the bedside of hospitalized patients comes from pediatrics.^33,34^ Parent caregivers play a critical role in the care of children who are hospitalized. Caregivers’ active engagement in children’s care improves shared decision-making, satisfaction, and identification of medication issues and reduces missingness in the medical record.^35,36,37^ Furthermore, it can improve outcomes such as increased child comfort, lower risk of adverse events, and shorter length of stay.^38^ However, many caregivers are unable to be present with their child in the hospital because of complex social, financial, and emotional factors.^39^ Barriers leading to parental caregiver distress include transportation difficulties, financial instability, and disrupted work.^39^

Competing demands can broadly affect those balancing multiple caregiving responsibilities. This is particularly an issue when distance to the hospital is an issue^26^ and has been noted as more pronounced among adolescents and children with public insurance who were less likely to have a caregiver present during hospitalizations.^33^ Not having a caregiver present is associated with increased readmission, opiate prescription, and adverse events.^33^ Thus, the FCCI includes long-distance work commute and childcare responsibilities that both affect time availability.

The role for family caregivers for acutely hospitalized adults is analogous to the role of parent caregivers for children. Being at the bedside during hospitalizations may be especially important when acute illness clouds the patient’s cognition. One study called this phenomenon *posthospital syndrome*, which is an acquired, transient confusion not limited to adults with dementia that can happen to many adult and older patients and start during the hospitalization.^40,41^ Posthospital syndrome is attributable to a combination of factors, including sleep disturbance, pain, the stress of dealing with unknown people, and the cognition-altering adverse effects of medications, and bedside caregivers familiar to the patient can help manage confusion and prevent complications.

Recognizing this, the RAND Corporation has developed a framework for integrating family caregivers into the health care team and for developing mutually supportive caregiver, patient, and physician relationships.^3^ They underscore the value of synchronous communication in order to deliver and ascertain urgent information for timely decisionmaking.^3^ Synchronous communication happens in real time, typically in person, but also through telecommunication. However, telecommunication requires internet access, which is missing for for approximately 10% of the US population, particularly in adults aged 65 years and older, and rural residents.^42^ Furthermore, approximately 20% of the population and 40% of older adults lack high-speed broad band service, which is necessary for teleconferencing platforms such as Zoom.^43^ This digital divide further exacerbates health care disparities for those who are dependent on family caregivers.

The FCCI includes a domain of nativity and recent immigration, which includes not having a car, not speaking English at home, and not being a citizen. This is again consistent with the pediatric literature that has shown, for example, that Hispanic and Latino parent caregivers at a safety-net hospital experienced transportation difficulties, work disruptions, and difficulty navigating the health care system.^44^

### Limitations

Our study has several imitations. First, analyses were at the ZCTA level, where aggregate characteristics may obscure important differences in smaller, homogeneous, neighborhood units such as census-block groups.^8^ Second, we did not examine the association of the FCCI with actual caregiver engagement in the hospital. Third, the temporal mismatch between the ACS (2017 to 2021) and Dartmouth Atlas outcome data (2017 to 2018) in the validity analysis may have contributed to misspecification of the association between the FCCI and health service outcomes. Mitigating these issues, the FCCI uses nationally weighted data, thus minimizing bias and optimizing generalizability.

## Conclusions

In this cross-sectional study, we developed the FCCI, a theoretically consistent index of barriers to family caregiving that demonstrates good internal validity and association with health care outcomes. This index is an important development for the field of caregiver research because it can be used in quantitate the supporting role of caregivers on population-level, patient health outcomes. Future research should examine associations among FCCI scores, caregiver engagement with hospitals, caregiver well-being, and health outcomes.

## Notes

Supplement 1. eTable 1. Initial Candidate Variables from the American Community Survey (ACS) Used to Create Compositive Variables Included in the Family Caregiver Constraint Index eTable 2. Demographic Characteristics of American Community Survey 2017-2021 5-year estimates eTable 3. Kaiser’s Measure of Sampling Adequacy eTable 4. Initial Components and their Eigenvalues from Principal Components Analysis eTable 5. Principal Components Analysis Results eTable 6. Unadjusted and Adjusted Relationship between the Family Caregiver Constraint Index and the Structural Racism Effect Index eFigure 1. Scree Plot of Eigenvalues from the Principal Components Analysis eFigure 2. Histogram of the Standardized Family Caregiver Constraint Index eAppendix. Model Selection and FCCI Construction from Principal Component Analysis

Supplement 2. Data Sharing Statement
