# Supplementary material for: Development and Validation of a Family Caregiver Constraint Index
Source: JAMA Netw Open. 2026 May 28;9(5):e2615350. doi: 10.1001/jamanetworkopen.2026.15350 (PMC13220108; doi:10.1001/jamanetworkopen.2026.15350)
Supplement: Supplement 1. — eTable 1. Initial Candidate Variables from the American Community Survey (ACS) Used to Create Compositive Variables Included in the Family Caregiver Constraint Index eTable 2. Demographic Characteristics of American Community Survey 2017-2021 5-year estimates eTable 3. Kaiser’s Measure of Sampling Adequacy eTable 4. Initial Components and their Eigenvalues from Principal Components Analysis eTable 5. Principal Components Analysis Results eTable 6. Unadjusted and Adjusted Relationship between the Family Caregiver Constraint Index and the Structural Racism Effect Index eFigure 1. Scree Plot of Eigenvalues from the Principal Components Analysis eFigure 2. Histogram of the Standardized Family Caregiver Constraint Index eAppendix. Model Selection and FCCI Construction from Principal Component Analysis [file jamanetwopen-e2615350-s001.pdf]

Supplemental Online Content

Tjia J, Asiedu E, Baek J, et al. Development and validation of a family caregiver constraint index. *JAMA Netw Open.* 2026;9(5):e2615350. doi:10.1001/jamanetworkopen.2026.15350

eTable 1. Initial Candidate Variables from the American Community Survey (ACS) Used to Create Compositive Variables Included in the Family Caregiver Constraint Index

eTable 2. Demographic Characteristics of American Community Survey 2017-2021 5-year estimates

eTable 3. Kaiser's Measure of Sampling Adequacy

eTable 4. Initial Components and their Eigenvalues from Principal Components Analysis

eTable 5. Principal Components Analysis Results

eTable 6. Unadjusted and Adjusted Relationship between the Family Caregiver Constraint Index and the Structural Racism Effect Index

eFigure 1. Scree Plot of Eigenvalues from the Principal Components Analysis

eFigure 2. Histogram of the Standardized Family Caregiver Constraint Index

eAppendix. Model Selection and FCCI Construction from Principal Component Analysis

This supplemental material has been provided by the authors to give readers additional information about their work.

© 2026 Tjia J et al. *JAMA Network Open*.

eTable1. Initial Candidate Variables from the American Community Survey (ACS) Used to Create Compositive Variables Included in the Family Caregiver Constraint Index

Initial ACS Variables Corresponding to Theoretical Family Caregiver Constraint Domains (n=29)	Composite Variables Used in Creation of the Family Caregiver Constraint Index (n=13)
Place of work – Worked outside county of residence	Work out of county
Place of work – Worked outside place of residence	Work outside of home
Work Status – Families – Two or more worker in past 12 months	Working persons
No vehicle available	No car
Means of Transportation to Work – Public Transport (excluding taxi)	Public transport use
Travel time to work – 60 mins or more	Long travel time to work
Household – With children under 18 years – Married couple family<br>Household – With children under 18 years – Other family<br>Household – With children under 18 years – Other family – Male householder, no spouse present<br>Household – With children under 18 years - Other family – Female household, no spouse present<br>Household – With children under 18 years – Nonfamily households	With children households
Has a computer without an internet subscription<br>No computer	Teleconference inability
Speaks a language other than English at home	Language other than English
Foreign born – Not citizen	Noncitizen

© 2026 Tjia J et al. *JAMA Network Open*.

• Household receiving food stamps	Food stamps use
• Monthly housing cost as percentage of household income – less than $20,000 – 30% or more<br>• Monthly housing cost as percentage of household income – $20,000 to $34,999 – 30% or more<br>• Monthly housing cost as percentage of household income – $35,000 to $49,999 – 30% or more<br>• Monthly housing cost as percentage of household income – $50,000 to $74,999 – 30% or more<br>• Monthly housing cost as percentage of household income – $75,000 or more – 30% or more<br>• Monthly housing cost as percentage of household income – Zero or negative income	Burden of monthly housing cost
• Household income in past 12 month – less than $5,000<br>• Household income in past 12 month - $5,000 to $9,999<br>• Household income in past 12 month - $10,000 to $14,999<br>• Household income in past 12 month - $15,000 to $19,999<br>• Household income in past 12 months - $20,000 to $24,999<br>• Household income in past 12 months - $25,000 to $34,999<br>• Household income in past 12 months - $35,000 to $49,999	Low income

**The 13 composite variables used in the principal components analysis were created from 29 ACS variables that were classified as a resource or burden in terms of effect on the time available for a family caregivers to be synchronously present at the hospital bedside.*

© 2026 Tjia J et al. *JAMA Network Open*.

eTable 2. Demographic Characteristics of American Community Survey 2017-2021 5-year estimate

	Total Population	% of Total Population
	329,725,481	100%
**Sex**		
Female	166,518,866	50.5%
Male	163,206,615	49.5%
**Age Group**		
under 19 years	82,998,739	25.2%
20 to 34 years	66,935,367	20.3%
35 to 44 years	42,441,883	12.9%
45 to 54 years	41,631,458	12.6%
55 to 64 years	42,829,413	13.0%
65 to 74 years	31,590,619	9.6%
75 to 84 years	14,998,214	4.5%
85 years and over	6,299,788	1.9%
**Race and Ethnicity**		
Hispanic or Latino	60,806,969	18.4%
Not Hispanic or Latino	268,918,512	
American Indian and Alaska Native alone	19,36,842	0.6%
Asian alone	18,554,697	5.6%
Black or African American alone	40,196,302	12.2%
Native Hawaiian and Other Pacific Islander alone	555,712	0.2%
Some other race alone	1,208,267	0.4%
Two or more races	104,456,322	3.2%
White alone	196,010,370	59.4%

© 2026 Tjia J et al. *JAMA Network Open*.

Source: DP05, 2021: ACS 5-Year Estimates Data Profiles, https://data.census.gov/table/ACSDP5Y2021.DP05

© 2026 Tjia J et al. *JAMA Network Open*.

eTable 3. Kaiser's Measure of Sampling Adequacy

Candidate Variable	Measure of Sampling Adequacy
Teleconference Inability	0.65
Public Transportation	0.64
No Care	0.61
Work Out of County	0.60
Work Out of Home	0.68
Long Travel Time	0.43
Language Other than English	0.59
Working Persons	0.78
Non-Citizen	0.60
Food Stamp Use	0.69
Household with Children	0.43
Low Income	0.61
Housing Burden	0.64
**Overall**	**0.62**

© 2026 Tjia J et al. *JAMA Network Open*.

eTable 4. Initial Components and their Eigenvalues from Principal Components Analysis

Factor	Eigenvalue	Difference	Proportion	Cumulative
Component 1	2.44	0.18	0.19	0.19
Component 2	2.26	0.95	0.17	0.36
Component 3	1.31	0.07	0.10	0.46
Component 4	1.24	0.19	0.10	0.56
Component 5	1.05	0.14	0.08	0.64

© 2026 Tjia J et al. *JAMA Network Open*.

eTable 5. Principal Components Analysis Results

	5 factors		4 factors	
**Component name**	**Dimension name**	**Loading**	**Dimension name**	**Loading**
Inability to teleconference	Income constraint	71	Income constraint	70
Two or more working persons in household	Income constraint	-60	Income constraint	-58
Food stamps use	Income constraint	65	Income constraint	70
Low income	Income constraint	88	Income constraint	88
Burden of monthly housing cost	Income constraint	53	Income constraint	52
Public transport use	Transport ation	77	Recent immigration	76
No car	Transport ation	83	Recent immigration	64
Language other than English at home	Recent immigration	85	Recent immigration	69
Noncitizen	Recent immigration	85	Recent immigration	72
Work outside the home	Recent immigration	49	Work and childcare	62
Household with children	Childcare responsib ilities	90	Work and childcare	68
Work out of	Long commute	75	Long commute	76

© 2026 Tjia J et al. *JAMA Network Open*.

county	to work		to work	
Long travel time to work	Long commute to work	81	Long commute to work	73

© 2026 Tjia J et al. *JAMA Network Open*.

eTable 6. Unadjusted and Adjusted Relationship between the Family Caregiver Constraint Index and the Structural Racism Effect Index (SREI)

**Unadjusted Model**			
	Estimate	95% CI	p-value
(Intercept)	-0.38	-0.39, -0.37	<0.001
ZCTA SREI	0.11	0.10, 0.12	<0.001
**Adjusted Model**			
	Estimate	95% CI	p-value
(Intercept)	-0.14	-0.29, -0.01	0.076
ZCTA SREI	0.10	0.08, 0.11	<0.001
% ZCTA Male	-0.01	-0.01, -0.008	<0.001
% ZCTA Age 65 or older	-0.0003	-0.002, 0.001	0.67
% ZCTA Hispanic or Latino	0.04	0.03, 0.04	<0.001
% ZCTA Black or African American	0.01	0.01, 0.01	<0.001
% ZCTA Asian	0.03	0.03, 0.03	<0.001
% ZCTA Other	-0.002	-0.005, 0.002	0.34

ZCTA: Zip code tabulation area. SREI: Structural Racism Effect Index. CI: confidence interval.
The Structural Racism Effect Index is based on publicly available data on forty-two variables within nine domains affected by structural racism: built environment, criminal justice, education, employment, housing, income and poverty, social cohesion, transportation, and wealth. Higher scores indicate greater disadvantage.[29]
Unit of analysis is the Zip Code Tabulation Area. Analysis uses N= 32,064 ZTCAs
Other includes American Indian and Alaska Native alone, Native Hawaiian and Other Pacific Islander alone, some other race alone, two or more races.

© 2026 Tjia J et al. *JAMA Network Open*.

eFigure 1. Scree Plot of Eigenvalues from the Principal Components Analysis

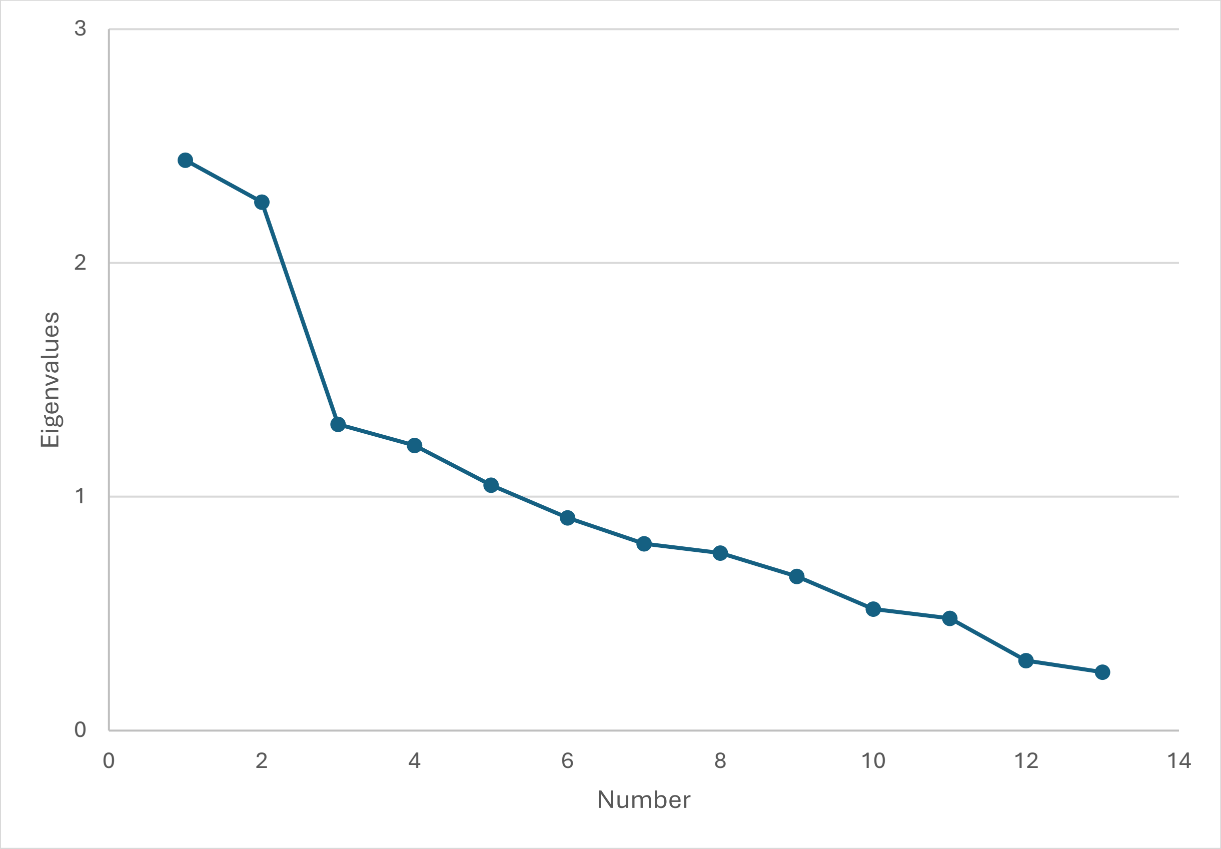

Legend. Scree plot of Eigenvalues with associated number of components

-

© 2026 Tjia J et al. *JAMA Network Open*.

eFigure 2. Histogram of the standardized Family Caregiver Constraint Index (FCCI)

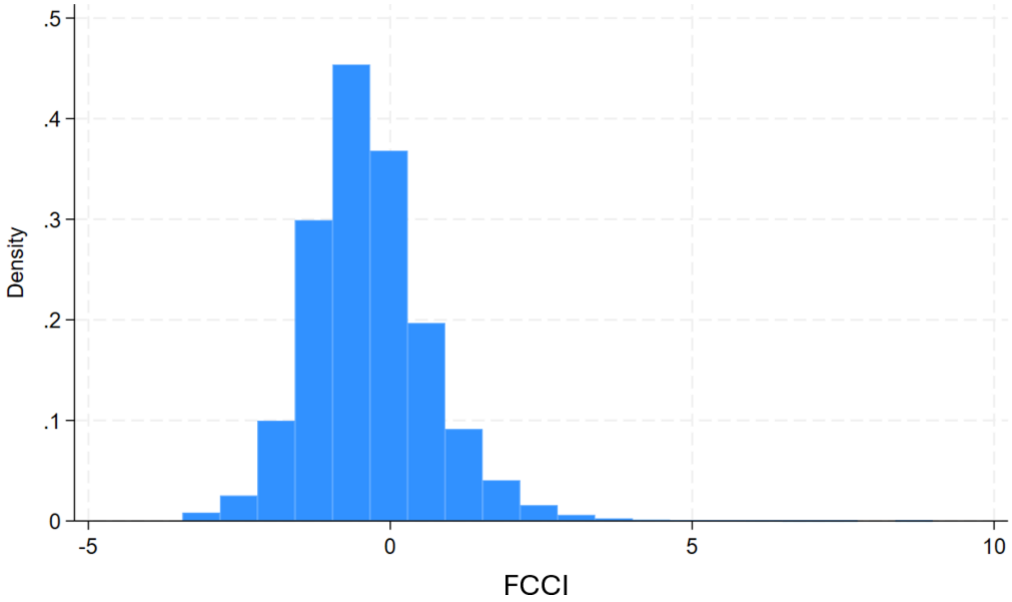

Legend. Histogram showing density of FCCI scores. The scores ranges from -3.44 to 8.98, with 92% of Zip Code Tabulation Areas in the United States having scores that fall between – 2 and +2.

© 2026 Tjia J et al. *JAMA Network Open*.

eAppendix. Model Selection and FCCI Construction from Principal Component Analysis

We used Eigenvalues and scree plots to inform factor structure and determine the number of domains to retain. We sorted items based on factor loadings and suppressed items with a loading ≤0.40. For each factor derived from the PCA, we looked at the composite variables that loaded >40 on that factor. When a composite variable loaded highly (> 0.40) on multiple factors, we put it with the factor on which it loaded highest. For each factor, we summarized the % of all the composite variable that loaded highest on that factor to derive sub-index scores, and summed these at the ZCTA level to form a raw summary score, S, which we then weighted to create a standardized FCCI (with mean = 0 and standard deviation = 1), as follows. In computing the mean and standard deviation we weighted each ZCTA by its total population so that each United States resident would contribute equally. That is, in ZCTA "i," let x_i and w_i be, respectively, the value of a variable x and the total population in that ZCTA. Then the mean of x is $\mu_x = \frac{\sum wixi}{\sum wi}$ and its standard deviation is $\sigma_x = \sqrt{\frac{\sum wi(xi - \mu x)^2}{\sum wi}}$. From these, for ease of interpretation, we standardize each x variable and the summary FCCI: $z_{ix} = \frac{xi - \mu x}{\sigma x}$ and $z_i = \frac{S - \mu S}{\sigma S}$.

© 2026 Tjia J et al. *JAMA Network Open*.
