# Supplementary material for: Development and Validation of a Family Caregiver Constraint Index
Source: JAMA Netw Open. 2026 May 28;9(5):e2615350. doi: 10.1001/jamanetworkopen.2026.15350 (PMC13220108; doi:10.1001/jamanetworkopen.2026.15350)
Supplement: Supplement 2. — Data Sharing Statement [file jamanetwopen-e2615350-s002.pdf]

Data Sharing Statement

Tjia. Development and Validation of a Family Caregiver Constraint Index. *JAMA Netw Open*. Published May 28, 2026. doi:10.1001/jamanetworkopen.2026.15350

Data
Data available: Yes
Data types: Data (not involving human participants)
How to access data: Data sets from the American Community Survey and Dartmouth Health Atlas will be available
When available: With publication

Supporting Documents
Document types: None

Additional Information
Who can access the data: Anyone requesting data
Types of analyses: Data file with FCCI geographically linked to zip code tabulation areas in the US
Mechanisms of data availability: Data will be made available without investigator support
